# A systematic review of epinephrine stability and sterility with storage in a syringe

**DOI:** 10.1186/s13223-019-0324-7

**Published:** 2019-02-21

**Authors:** Hannah G. Parish, Jacquelyn R. Morton, Julie C. Brown

**Affiliations:** 1 Seattle Children’s Hospital, 4800 Sandpoint Way NE, Seattle, WA 98105 USA; 2 Department of Pediatrics, Division of Emergency Medicine, University of Washington, Seattle, WA 98195 USA

**Keywords:** Epinephrine, Adrenaline, Syringe, Prefilled, Stability, Sterility, Storage, Time, Allergy, Anaphylaxis

## Abstract

**Background:**

Epinephrine is a lifesaving medication in the treatment of anaphylaxis. Epinephrine auto-injectors are the preferred method of epinephrine administration, but are not universally available or affordable. Little is known about the effects on epinephrine when it is drawn up in advance and stored as prefilled syringes.

**Objective:**

To study the stability and sterility of epinephrine when stored in syringes.

**Methods:**

We searched Embase, Medline, and Web of Science in June 2016 for all studies of epinephrine stored in syringes in concentrations between 0.1 and 1 mg/mL that measured epinephrine stability and/or sterility over time, regardless of date published or language.

**Results:**

Three studies were included, one testing two concentrations of epinephrine. Only one study tested epinephrine 1 mg/mL, the concentration clinically relevant for intramuscular use during anaphylaxis. Neither this study nor the one study testing 0.7 mg/mL epinephrine found significant degradation after 56 and 90 days, respectively. One of the two studies testing epinephrine at a concentration of 0.1 mg/mL found significant degradation by 14 days; the other found no degradation up to 168 days. Two studies tested for bacterial growth, with none detected after 28 and 90 days, respectively. One study tested for fungal growth, with none detected after 90 days.

**Conclusions:**

Limited evidence suggests that syringes filled with 1 mg/mL epinephrine are stable and sterile for 90 days. More research is needed testing the duration of stability and sterility of prefilled syringes with the 1 mg/mL concentration most commonly used in anaphylaxis, testing more extensively in different storage conditions and across a wider range of marketed syringe brands.

## Background

Epinephrine is the only first-line therapy for anaphylaxis. Guidelines recommend that individuals with life-threatening allergies carry one or more epinephrine auto-injectors (EAIs) in case of contact with their allergen [1, 2]. However, in many parts of the world EAIs are unavailable or not readily accessible [3], and rising costs of EAIs, particularly in the United States, are creating a financial barrier for both private consumers and health care systems [4–7]. Patients may seek alternatives to the EAIs they need but cannot access or afford [4–7]. In some countries, insurance plans cover epinephrine in vials provided with syringes for self-preparation, but not auto-injector devices [8]. In pre-hospital and hospital settings, the high costs of EAIs has resulted in increased use of epinephrine in vials and ampules, drawn up into a syringe either in advance or at the time of use [9–11].

Parents and caregivers have difficulty drawing up epinephrine from a vial into a syringe accurately or reliably [12], as may patients themselves. Preparing and storing epinephrine in syringes in advance may decrease errors as well as ensure timely administration, compared with drawing up epinephrine at the time of need. This systematic review was performed to identify and evaluate all published studies on the stability and sterility of epinephrine when drawn up and stored in prefilled syringes.

## Methods

The databases Embase.com, Medline in Ovid, and Web of Science were searched in June 2016 for relevant studies. This literature search was conducted following PRISMA guidelines [13]. No limits were applied to the searches except for the time constraints of the databases themselves. Medline contains literature from 1946 to the present, Embase.com from 1974 on, and Web of Science from 1985 on. In Medline and Embase, appropriate Medical Subject Headings (MeSH) or Emtree headings were used, respectively, in addition to keywords and text words. Medline and Embase were searched by our experienced librarian (J.R.M.) for the keywords *epinephrine* or *adrenalin* in combination with *syringe*, *“pre*-*filled”*, or *prefilled*. Web of Science was searched for the keyword syringe* in combination with epinephrin* or adrenalin* in the title. All studies captured in the search were imported into an electronic reference manager for management and review. Duplicates were removed using the “Find Close Duplicates” function in the reference manager, which was checked manually to ensure that no studies were incorrectly removed. Duplicates that were not identified electronically due to slight formatting differences between databases were manually removed during first review and accounted for in the total number of duplicates. Potentially relevant articles in other languages were translated into English using Google Translate. All potentially relevant English language studies were searched forward in Web of Science in order to find newer sources in which the original ones were cited. The references of all potentially relevant English language studies were reviewed to search for additional relevant studies.

### Inclusion criteria

Included studies assessed epinephrine in clinically relevant concentrations between 0.1 and 1 mg/mL that had been drawn up into prefilled syringes. In the United States and Canada, epinephrine as an anaphylaxis drug is commercially available in EAIs in two concentrations, 1 mg/mL and 0.5 mg/mL [14, 15]. When epinephrine is administered intramuscularly during anaphylaxis using a needle and syringe, as is often done in the hospital setting, it is typically drawn up from a vial or ampule with a 1 mg/mL concentration. Epinephrine at a concentration of 0.1 mg/mL was also considered clinically relevant due to its use as a cardiac resuscitation drug. Included studies reported epinephrine concentration at each time point as any of: (a) the percent of the labeled dose, based on manufacturer’s standards, (b) the relative percent of the initial dose measured at the start of the study, or (c) the relative percent of a control dose. Included studies described storage of epinephrine in accordance with commercial manufacturer guidelines, which recommend 1 mg/mL epinephrine ampules, vials, and auto-injectors be stored at controlled room temperature (defined as 20 °C to 25 °C, with a mean kinetic temperature not more than 25 °C and excursions permitted between 15 and 30 °C) and protected from light [14–21]. Included studies contained epinephrine as the only active ingredient in the syringe (other than drug stabilizers, such as sodium bisulfite or sodium metabisulfite; simple buffers, such as hydrochloric acid; and salts such as sodium chloride); therefore, studies that tested a mixed solution of epinephrine and another active ingredient (e.g. lidocaine with epinephrine) were excluded. Studies were excluded if the epinephrine tested exceeded the expiration date prior to or during the course of the study.

One investigator (H.G.P.) reviewed all study titles and abstracts to identify potentially relevant studies. These resulting studies were reviewed by two investigators (H.G.P. and J.C.B.), who each read the full text independently to determine eligibility. The rationale for excluding each potentially relevant study was discussed to confirm consistent reasoning. Any discrepancies in inclusion were resolved through discussion. Data including measured epinephrine concentrations at each time point, results of statistical tests, notes on other degradation tests or solution appearance, results of sterility testing, sample sizes for each test, timing of each test, and characteristics of the epinephrine source, syringe and needle, syringe preparation conditions, and storage conditions were collected from each included study independently by two investigators (H.G.P and J.C.B.). Any discrepancies in data collection were resolved through discussion and re-examination of the study. When relevant study details were missing from an included study, attempts were made to contact the corresponding author of the study to obtain these details. The concentration of epinephrine remaining at each time point was recorded for all studies as either the percentage of labeled dose of the test sample or the mean percentage relative to an initial dose, based on which information was available.

## Results

**Fig. 1:**
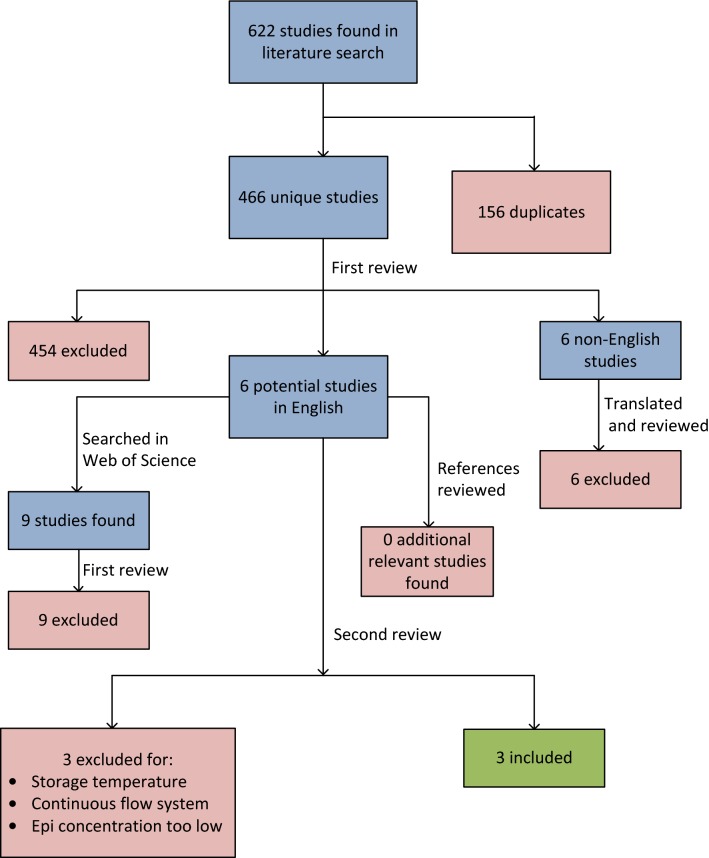
Flowchart of study selection

Literature searches resulted in 209 studies from Medline, 396 studies from Embase, and 17 studies from Web of Science. Of these, 156 studies were duplicates between the databases, yielding 466 unique studies. There were 454 studies excluded on first review based on titles, abstracts, or articles that clearly indicated non-relevance, leaving six potentially relevant studies published in English [11, 22–26] and six published in other languages. The six English language studies were then searched forward in Web of Science, yielding nine new studies. All nine were excluded on first review. The references of the six English language studies were then reviewed, yielding no additional relevant studies. The six potentially relevant English articles underwent further review, and three of these studies were determined to be eligible for inclusion (Fig. 1) [11, 22, 24]. Of the three studies excluded at this stage, one was excluded because the epinephrine was stored in a continuously infusing syringe pump system rather than a simple syringe, which was excluded due to the mobile nature of that system [23]. Another study was excluded because the syringes were stored in incubators at 38 °C, which is well above the recommended storage range for epinephrine [25]. The third excluded study diluted the 1 mg/mL epinephrine to a 1 in 100 solution before storage in sterile plastic syringes, making the concentration of epinephrine stored too low for inclusion [26]. The six studies published in a different language (three in French [27–29], one in Dutch [30], one in Spanish [31], and one in Danish [32]) were translated from their respective languages into English using Google Translate and reviewed by two authors. None of these six studies were determined to be eligible for inclusion.

Ultimately, three studies were included in this review (Fig. 1) [11, 22, 24]. There were no differences to resolve regarding study eligibility or data abstraction. The characteristics of the included studies are shown in Table 1.

**Table 1:** Characteristics of included studies

Study	Epinephrine source concentration (mg/mL)	Epinephrine source container	Epinephrine source manufacturer and location	Storage syringe and needle type	Syringe manufacturer and location	Preparation/transfer conditions	Storage conditions	Varying conditions studied
Donnelly [22]	1	Ampule	Parke-Davis Limited; Scarborough, Ontario, Canada	10 mL sealed glass vial, or 10 mL plastic syringe with 18G needle attached and capped	Glass vials: Miles Incorporated; Spokane, Washington, United States Plastic syringes: Becton, Dickinson and Company (BD); Franklin Lakes, New Jersey, United States	Epinephrine diluted with sterile water to 0.1 mg/mL or 0.7 mg/mL before transfer to syringe or vial	Stored protected from light and at room temperature	Storage container: sealed glass vial vs plastic syringe with needle attached
Kerddonfak [24]	1	Ampule	Government Pharmaceutical Organization; Bangkok, Thailand	Disposable plastic 1 mL syringe with 23G needle attached and recapped	Nipro; Osaka, Japan	1 mL of epinephrine transferred into syringe under either laminar flow hood or open air, then air bubbles removed from syringe	Stored in pencil box to protect from light, storage at ambient room temperature (26 ± 3 °C, measured at 8–9 a.m.)	Transfer conditions: laminar flow vs open air
Zenoni [11]	1	Ampule	Monico; Venice, Italy	1 mL Luer-lock polycarbonate latex-free syringe with no needle attached, sealed with a plastic cap	BD; Republic of Singapore	Epinephrine diluted with pyrogen-free water to 0.1 mg/mL and transferred into syringe in contamination controlled environment	Stored in heat-sealed light-proof plastic pocket, at room temperature (20–24 °C) or 2–8 °C	Storage temperature: 20–24 °C vs 2–8 °C

*vs* versus

The three included studies tested the epinephrine samples for degradation after different storage times, as shown in Table 2. In all three studies, there were multiple study arms being compared. In two cases, one arm of the study met inclusion criteria but the other did not. When this occurred, the study was included and the excluded arm was presented in Table 2 in italics, for comparison purposes.

**Table 2:** Stability of epinephrine in prefilled syringes

Study	Concentration of epinephrine (mg/mL)	Variable conditions	# of samples in study arm	Time period of storage (24 h days)	Mean percent of initial (or labeled^a^) dose remaining (%)	Significance test used	Significant degradation of epinephrine concentration? (yes/no)	Notes
Donnelly [22]	0.1	Stored in plastic syringe	4^b^	7	96.0 ± 1.01	Clinical significance: < 90% of mean initial dose remaining	No	No other signs of degradation noted; all solutions remained clear and colorless; needles capped
			4^b^	14	88.5 ± 1.29		Yes	
			4^b^	28	86.2 ± 1.40		Yes	
			4^b^	56	79.5 ± 2.06		Yes	
		*Stored in glass vial*	*4* ^b^	*7*	*94.2 *±* 2.60*		*No*	
			*4* ^b^	*14*	*87.4 *±* 1.36*		*Yes*	
			*4* ^b^	*28*	*83.6 *±* 1.22*		*Yes*	
			*4* ^b^	*56*	*76.7 *±* 1.95*		*Yes*	
	0.7	Stored in plastic syringe	4^b^	7	98.5 ± 1.98		No	
			4^b^	14	96.8 ± 1.38		No	
			4^b^	28	94.0 ± 3.69		No	
			4^b^	56	94.6 ± 6.47		No	
		*Stored in glass vial*	*4* ^b^	*7*	*99.2 *±* 0.27*		*No*	
			*4* ^b^	*14*	*98.3 *±* 0.55*		*No*	
			*4* ^b^	*28*	*98.3 *±* 1.06*		*No*	
			*4* ^b^	*56*	*97.8 *±* 1.42*		*No*	
Kerddonfak [24]	1	Prepared under laminar flow hood	20	30	101.40^a^	Clinical significance: < 90% or > 110% of labelled dose remaining	No	No other signs of degradation noted in samples; norepinephrine not detected in any sample; brown particles found at needle cap in some syringes; solutions were pink-brown at 4 months
			20	60	102.68^a^		No	
			20	90	101.19^a^		No	
		Prepared in open air	20	30	101.36^a^		No	
			20	60	99.31^a^		No	
			20	90	101.09^a^		No	
Zenoni [11]	0.1	Storage at room temperature (20–24 °C)	2^c^	7	90 ± 1.2	Statistical significance: one-way ANOVA; Clinical significance: < 90% of labelled dose remaining	Statistically yes (compared to refrigerated storage, p < 0.002); clinically no	No other signs of degradation noted: no degradation products detected, no changes in color or clarity visualized
			2^c^	14	91 ± 0.3			
			2^c^	28	91 ± 0.5			
			2^c^	56	98 ± 0.5			
			2^c^	168	96 ± 0.7			
		*Storage at 2*–*8* *°C*	*2* ^c^	*7*	*104 *±* 0.1*		*No*	
			*2* ^c^	*14*	*105 *±* 0.1*			
			*2* ^c^	*28*	*102 *±* 1.0*			
			*2* ^c^	*56*	*110 *±* 1.0*			
			*2* ^c^	*168*	*104 *±* 3.8*			

^a^Study measured percent of manufacturer’s labeled dose

^b^4 samples were obtained from 2 syringes or vials tested in duplicate

^c^2 samples were obtained from 1 syringe tested in duplicate

Only one study evaluated 1 mg/mL epinephrine, as would typically be used to treat anaphylaxis [24]. No significant epinephrine degradation was found in prefilled syringes at any time point up to the 3 months of storage studied.

One included study compared the stability of epinephrine stored in prefilled syringes between two different concentrations of epinephrine [22]. This study found that 0.7 mg/mL epinephrine remained stable for the 8-week duration of the study, whereas 0.1 mg/mL epinephrine showed clinically significant degradation by 14 days. The syringes were stored with 18G needles attached, which exposed the epinephrine in the needle to air during storage. Another included study evaluated 0.1 mg/mL epinephrine and found no clinically significant degradation at any point over the 24-week duration of the study [11]. In the latter study, the syringes were stored without needles, tightly sealed with a plastic cap, and protected inside a heat-sealed light-proof plastic pocket.

Two of the three included studies performed tests to determine the sterility of the prefilled syringe samples during the study period (Table 3) [11, 24]. Kerddonfak tested for gram-negative bacteria, gram-positive bacteria, and fungal cultures after 1, 2, and 3 months of storage and detected no growth in any sample, regardless of whether the samples were prepared under a sterile laminar flow hood or in open air. In a few syringes, some brown particles were found at the needle cap after storage. These particles were cultured and found to be neither bacterial nor fungal, and were hypothesized to be from a reaction between epinephrine and the air at the needle cap. Zenoni performed particulate testing, limulus amebocyte lysate (LAL) testing for the presence of endotoxin, and visual inspection of the solutions at time zero and after 4 weeks (timing and methods verified by personal communication) and all tests were negative.

**Table 3:** Sterility of epinephrine in prefilled syringes

Study	Concentration of epinephrine (mg/mL)	Type of epinephrine container used	Total # of samples tested	Sterility tests performed	Testing methods	Time at which sterility tested	Results
Donnelly [22]	0.1	Plastic syringe	None	None	N/A	N/A	N/A
		Glass vial					
	0.7	Plastic syringe					
		Glass vial					
Kerddonfak [24]	1	1-mL disposable syringe, prepared under laminar flow hood	6 syringes (2 per time point)	Tests for gram-negative bacteria, gram-positive bacteria, and fungal cultures	Syringes (1 mL each) tested for bacteria on blood agar incubated at 35 °C in air and for fungal cultures on brain heart blood agar incubated at 37 °C and sabouraud dextrose agar incubated at 37 °C	At each time point (after 1, 2, and 3 months of storage) for both conditions	No aerobic bacterial or fungal growths in any sample
		1-mL disposable syringe, prepared in open air	6 syringes (2 per time point)				No aerobic bacterial or fungal growths in any sample
Zenoni [11]	0.1	1-mL polycarbonate syringes	6 syringes (2 at time zero, 2 stored at room temperature, and 2 stored at 2–8 °C)	Tests for sterility, absence of bacterial endotoxin, and the rate of particle contamination	Particulate test according to European Pharmacopeia, limulus amebocyte lysate (LAL) test for the presence of endotoxin, visual examination of the solution	At time zero and after 4 weeks	All tests negative: bacterial contamination absent, endotoxin levels < 0.125 EU/mL, no visually detectable changes in color or clarity of solution, invisible particle count (< 10 μm and < 25 μm) below detectable limits

## Discussion

The incidence of allergies and anaphylaxis continues to increase worldwide, especially among children [33]. Epinephrine is a critical life-saving therapy for anaphylaxis and must be readily available to those at risk in order to reduce morbidity and mortality [1]. EAIs are the preferred administration method for epinephrine due to their relative ease of use and proven stability [1, 34]. EAIs contain epinephrine in a sealed container that protects the drug from exposure to oxygen and light, allowing the epinephrine to remain pharmacologically stable for at least 1 year after the device is produced. Some studies have indicated that epinephrine stored in EAIs may remain stable and clinically usable for much longer than 1 year, although results vary by device and between studies [35–37]. Auto-injector devices reduce errors by eliminating some of the variables of administering the drug, such as determining the correct dose [12, 38]. The EAI user does not have to see the needle prior to use, or be experienced in how to self-administer a medication using a needle and syringe, potentially reducing barriers to use [39].

The prospect of administering epinephrine safely without an EAI raises concerns among doctors and patient advocates, who believe it is more complicated to administer the correct dose safely using a syringe and needle [5]. However, patients and providers have had to consider this alternative in many parts of the world where EAIs are unavailable or cost-prohibitive. As of 2007, auto-injectors containing 0.3 mg of epinephrine were officially available in only 39 countries worldwide (20% of the 195 countries) [3]. EAIs with doses appropriate for infants were not available in any country at that time, although a 0.1 mg EAI with a shorter needle was recently marketed in the United States [40]. Even in areas where auto-injectors are distributed, they may not be accessible to all patients at risk of anaphylaxis. EAIs are potentially cost-prohibitive, particularly in the United States, where escalating EAI costs, rising insurance deductibles for many patients, and varying coverage of EAIs makes for a complicated system for consumers to navigate successfully [41]. The use of a syringe and needle to deliver epinephrine is an inexpensive alternative in these cases [7] and also allows for customizable doses and needle lengths, which may benefit patients for whom the standard needle length of an auto-injector is too short (resulting in subcutaneous injection) or too long (resulting in injection into bone) [42]. However, a patient or caregiver may be unable to quickly and correctly draw up a dose of epinephrine during the stress and time constraints of an anaphylactic reaction [12, 43].

Prefilled syringes can be prepared in advance, either in clinic or in a pharmacy, for the patient to take home. Transferring epinephrine from an ampule or vial into the syringe potentially exposes the epinephrine to oxygen, which could accelerate degradation. The medication also potentially risks contamination during transfer. Although evidence is limited, the results of this systematic review suggest that epinephrine prefilled syringes, in concentrations typically used in anaphylaxis, appear to be a viable alternative to EAIs. Under recommended storage conditions, 1 mg/mL epinephrine is stable in a syringe for at least 3 months [24] and 0.7 mg/mL epinephrine is stable in a syringe for at least 8 weeks [22]. An additional study by Rawas-Qalaji et al., excluded from this review based on prolonged high heat exposure, showed similar stability of epinephrine. In this study, syringes containing 1 mg/mL epinephrine and stored at 38 °C still delivered at least 90% of the label dose after 2 months of storage in low humidity or 3 months in high humidity [25]. These results are especially notable because high storage temperatures are expected to accelerate degradation of epinephrine compared to room temperature storage [44].

Donnelly’s study was the only one which compared the stability between two different concentrations of epinephrine stored in prefilled syringes [22]. This study found that 0.7 mg/mL epinephrine remained stable for the full 8 weeks of the study, while 0.1 mg/mL epinephrine showed clinically significant degradation by 14 days. These results suggest that higher concentrations of epinephrine are more stable in syringes over time. The stability of 1 mg/mL epinephrine in Kerddonfak’s study further supports this idea [24]. However, another study did not find any clinically significant degradation of 0.1 mg/mL epinephrine in prefilled syringes over 24 weeks [11], indicating that different storage conditions may have an impact on stability of epinephrine regardless of concentration.

Each study stored the epinephrine-filled syringes in a slightly different way, which may have contributed to the variation seen in the results. In Donnelly’s study, in which the 0.1 mg/mL epinephrine samples demonstrated significant degradation past 7 days of storage, capped needles were attached to the epinephrine prefilled syringes during storage. The needle allowed air exposure during storage that may have contributed to the degradation of epinephrine seen in this study [22]. In Zenoni’s study, which also tested 0.1 mg/mL epinephrine, the syringes were stored without needles attached, sealed with a plastic cap, and kept inside a sealed, light-proof plastic container. In this study, no significant degradation of epinephrine was detected over 24 weeks of storage [11]. This more rigorous storage process may have reduced air exposure and minimized the impacts of light, moisture, and oxygen on the degradation of epinephrine.

Zenoni additionally compared storage of 0.1 mg/mL epinephrine at room temperature to storage in a refrigerator (2–8 °C) [11]. Storing epinephrine prefilled syringes at 2–8 °C resulted in significantly higher concentrations of epinephrine compared to the syringes stored at room temperature over the course of the 24-week study, although neither group showed clinically significant degradation (epinephrine concentration below 90%) by 24 weeks. Although epinephrine is recommended for storage at room temperature in the United States, studies on epinephrine stored in cold temperatures, including freezing, have found no significant degradation, even up to a full year of storage [44].

Neither of the two studies testing for bacterial growth [11, 24] nor the one study testing for fungal growth [24] identified any contamination. In addition, another study demonstrated that prefilled syringes containing 1 mg/mL epinephrine remained sterile after storage in contaminated soil, whereas epinephrine ampules, needles, and syringes stored separately in the same contaminated soil before combining them for use resulted in contamination of six out of 10 solutions [45]. Prefilled syringes may offer a more sterile solution in the community than providing an ampule, needle, and syringe. However, the testing performed in these studies was on a very limited number of samples (Table 3) and only up to 3 months of storage, and therefore cannot be extrapolated to the long-term sterility of all epinephrine syringes. Any prefilled syringes distributed to patients or used in the hospital setting would need to be properly tested for sterility as would be expected for a commercial product distributed under national guidelines.

The idea of using prefilled syringes to treat anaphylaxis is not new: a paper published in 1975 describes kits containing antihistamine tablets and a preloaded syringe of epinephrine available by prescription [46]. While an epinephrine prefilled syringe was recently approved by the United States Food and Drug Administration and brought to market in clinical settings [47], general inaccessibility of affordable epinephrine devices has led to “off-label” use of epinephrine prefilled syringes in many allergists’ offices of the United States (personal communication), and in community and hospital environments around the world. In countries where diluted (0.1 mg/mL) solutions of epinephrine are unavailable, stock epinephrine may be routinely diluted and stored in syringes as ready-to-use preparations in the hospital setting [11]. While there are United States Pharmacopeia standards for compounded medications allowing only 1–3 days of storage (depending on conditions) after compounding [48, 49], we are unaware of any industry standards specific to drawing up epinephrine or other medications without compounding or dilution, and intended for intramuscular use. There have been reports of infections associated with medications stored in syringes and then delivered intravenously [50], but we are unaware of any infections associated with intramuscular use of syringes. There has, however, been one reported Clostridial infection following an EpiPen injection in a thigh [14], suggesting that this potential exists.

Prefilled syringes are inexpensive and allow the provider to tailor the dose and needle length to meet the patient’s needs. However, prefilled syringes might be most valuable in pre-hospital and hospital settings, where the high costs of EAIs has resulted in increased use of epinephrine in syringes, most commonly drawn up from ampules or vials at the time of use [9, 10, 51]. Epinephrine prefilled syringes may be a better alternative to drawing up epinephrine at the time of need, as drawing up doses using an ampule and syringe under time pressure is prone to errors even among medical professionals [12]. Thus, stocking prefilled syringes in the medical setting, ideally with a range of weight-based doses, might minimize errors and potentially reduce the time taken to get epinephrine to a patient experiencing anaphylaxis. Storing resuscitation medications in color-coded prefilled syringes has been shown to reduce administration time and errors among emergency medical professionals [52].

To date, there is no definitive evidence to suggest how long 1 mg/mL epinephrine prefilled syringes are stable or sterile, as the current research has only indicated stability and sterility for up to 3 months. This potentially limited time to expiration may be problematic particularly in the community setting, where patients might not reliably change out their medication on time. Prefilled syringes also require protection from light exposure and must be stored in a container that prevents the syringe plunger from being unintentionally depressed prior to use, limitations that must be addressed before prefilled syringes can be a feasible storage mechanism for ready-to-use epinephrine.

### Limitations of the research

In order to find every potential study, we did not limit the results of the literature search to English. We used Google Translate to translate the six non-English studies found in the search. We did not verify that our translations were correct with someone fluent in each language, so we may have missed pertinent information from these six studies. However, we do not believe that this is probable, given that none of these studies appeared relevant or remotely likely to be eligible for inclusion in this review.

In one study, mean storage room temperatures measured between 8 and 9 a.m. were 26 ± 3 °C [24]. Daily temperatures could have exceeded recommended room temperature ranges (20–25 °C, with excursions allowed up to 30 °C but a 24-h mean of no more than 25 °C) [14–21]. Since no 24-h mean temperature was provided in the study or was available after personal communication with the author, we decided that we did not have sufficient evidence that the mean daily temperatures exceeded those allowed under manufacturer recommended storage temperatures, and concluded that this study was eligible for inclusion. Fortunately, the possible heat exposure in this study does not appear to have impacted study conclusions. If the epinephrine had been exposed to excessive heat during the study, it would have been expected to cause increased degradation of epinephrine, which was not seen.

All three of the authors we attempted to contact replied (Benjaponpitak, Invernizzi, Donnelly), and two (Invernizzi and Donnelly) were able to provide additional information. However, no study was excluded based on lack of information.

Only three studies were eligible for this systematic review, and only one eligible study tested epinephrine at the 1 mg/mL concentration that would be appropriate in an epinephrine prefilled syringe for use in anaphylaxis [24]. Only a small range of different syringe brands were tested; results could vary by syringe type and storage conditions. Only two studies tested samples for bacterial contamination and one for fungal contamination. One of these studies tested 12 syringes total [24] and the other tested 6 syringes total [11]. These small sample sizes may have been insufficient to detect rare contamination events. Therefore, the implications of this systematic review are limited due to the small number of relevant studies.

## Conclusions

EAIs are the preferred method for epinephrine administration during an anaphylactic event, and the ideal solution to their current inaccessibility is making the devices affordable and widely available in a range of doses and needle lengths that meet the needs of all patients with allergies. Epinephrine prefilled syringes for intramuscular delivery might offer an alternative when EAIs are not an option. They might also have a valuable role in prehospital and hospital care. Prefilled syringes have the potential to reduce dosing errors and decrease time to drug administration when compared with the traditional ampule, needle, and syringe method. Commercially-produced prefilled syringes may offer an even better alternative, if they remain affordable.

Syringes filled with 1 mg/mL epinephrine, the concentration typically used for anaphylaxis, have been shown by one study to be stable and sterile for at least 3 months [24]. One study has demonstrated that syringes containing 0.7 mg/mL epinephrine remain stable for at least 8 weeks [22], while the two studies examining 0.1 mg/mL epinephrine syringes show varied results [11, 22]. No study testing for sterility has found bacterial or fungal contamination of the prefilled syringes after storage for up to 3 months [11, 24], although this testing was limited to a small number of samples and may not reflect long-term sterility of all epinephrine prefilled syringes. More research is needed on the duration of stability and sterility of 1 mg/mL epinephrine prefilled syringes, as only one study thus far has examined this concentration of epinephrine that is relevant for the intramuscular treatment of anaphylaxis. If used, epinephrine prefilled syringes should be stored with sealed caps in light-blocking containers that prevent the syringe plungers from depressing, either at room temperature or refrigerated. They should be stored along with an appropriately sized needle in a sterile package, ideally along with alcohol swabs and gauze. As EAIs remain unaffordable and inaccessible for many patients, there may be an increasing place for these epinephrine prefilled syringes in the treatment of anaphylaxis.

## Abbreviations

EAI: epinephrine auto-injector
EMS: emergency medical services
